# Phase variable DNA repeats in *Neisseria gonorrhoeae* influence transcription, translation, and protein sequence variation

**DOI:** 10.1099/mgen.0.000078

**Published:** 2016-08-25

**Authors:** Marta A. Zelewska, Madhuri Pulijala, Russell Spencer-Smith, Hiba-Tun-Noor A. Mahmood, Billie Norman, Colin P. Churchward, Alan Calder, Lori A. S. Snyder

**Affiliations:** ^1^​ School of Life Sciences, Pharmacy, and Chemistry, Kingston University, Penrhyn Road, Kingston upon Thames, UK

**Keywords:** gonococcus, phase variation, C-terminal variation, homopolymeric tract, simple sequence repeats

## Abstract

There are many types of repeated DNA sequences in the genomes of the species of the genus *Neisseria*, from homopolymeric tracts to tandem repeats of hundreds of bases. Some of these have roles in the phase-variable expression of genes. When a repeat mediates phase variation, reversible switching between tract lengths occurs, which in the species of the genus *Neisseria* most often causes the gene to switch between on and off states through frame shifting of the open reading frame. Changes in repeat tract lengths may also influence the strength of transcription from a promoter. For phenotypes that can be readily observed, such as expression of the surface-expressed Opa proteins or pili, verification that repeats are mediating phase variation is relatively straightforward. For other genes, particularly those where the function has not been identified, gathering evidence of repeat tract changes can be more difficult. Here we present analysis of the repetitive sequences that could mediate phase variation in the *Neisseria gonorrhoeae* strain NCCP11945 genome sequence and compare these results with other gonococcal genome sequences. Evidence is presented for an updated phase-variable gene repertoire in this species, including a class of phase variation that causes amino acid changes at the *C*-terminus of the protein, not previously described in *N. gonorrhoeae*.

## Data Summary

1. Sequence data for *Neisseria gonorrhoeae *strains investigated are available in GenBank under the following accession numbers: FA1090 (NC_002946.2; url - http://www.ncbi.nlm.nih.gov/nuccore/NC_002946.2); NCCP11945 (NC_011035.1; url – http://www.ncbi.nlm.nih.gov/nuccore/NC_011035.1; & CP001050.1; url - http://www.ncbi.nlm.nih.gov/nuccore/CP001050.1); MS11 (NC_022240.1; url -http://www.ncbi.nlm.nih.gov/nuccore/NC_022240.1); FA19 (NZ_CP012026.1; url – http://www.ncbi.nlm.nih.gov/nuccore/NZ_CP012026.1); FA6140 (NZ_CP012027.1; url – http://www.ncbi.nlm.nih.gov/nuccore/NZ_CP012027.1); 35/02 (NZ_CP012028.1; url – http://www.ncbi.nlm.nih.gov/nuccore/NZ_CP012028.1). These were accessed on the 15^th^ of April 2016 for use in this study.
2. Sequence data were assessed via BLAST interrogation of the nr database restricted to the *N. gonorrhoeae* species (url – http://www.ncbi.nlm.nih.gov/).
3. Genome resequencing data for *N. gonorrhoeae* strain NCCP11945 has been deposited under BioProject PRJNA322254 (url – http://www.ncbi.nlm.nih.gov/bioproject/322254).
4. Genome resequencing data was assessed via Galaxy (url – usegalaxy.org).

## Impact Statement

Phase variation plays a vital role in the ability of *Neisseria gonorrhoeae *to adapt to the various niche environments encountered. Through stochastic switching in the expression of key genes and regulatory systems, mediated by simple sequence repeats, the population of bacteria are diverse and readily able to survive in the face of selective pressures. Not all simple sequence repeats within the genome mediate phase variation. Previous investigations have sought to define the phase-variable repertoire of the species of the genus *Neisseria* and have identified a large number of candidates using a small number of genome sequences. With the availability of more genome sequence data and additional experimental data, we have refined the original repertoire to include those most likely to be phase-variable in *N. gonorrhoeae*. As these genes are important for survival, their definition as phase-variable is important for understanding pathogenesis and for potential future therapies. The advent of high-throughput sequencing has the potential to reveal additional cases of within-strain variations in repeat tracts, supporting phase-variable candidacy of genes.

## Introduction

In *Neisseria gonorrhoeae*, the causative agent of gonorrhoea, DNA repeats are intimately linked to the biology of the organism. *N. gonorrhoeae*, and the closely related bacterial species *Neisseria meningitidis*, undergo phase-variable stochastic switching of gene expression for several surface structures, contributing to antigenic variation and immune evasion as well as niche adaptation in the course of infection (Bhat *et al.*, 1991; Moxon *et al.*, 2006; Carbonnelle *et al.*, 2009; Srikhanta *et al.*, 2009; Omer *et al.*, 2011). Phase variation is mediated by simple sequence repeats associated with genes. In the species of the genus *Neisseria* the vast majority contain homopolymeric tracts within the coding sequences (Snyder *et al.*, 2001).

Comparative sequence analysis between a single *N. gonorrhoeae *and several *N. meningitidis *genome sequences identified over 100 potentially phase-variable genes (Snyder *et al.*, 2001), some of which have later been demonstrated to be phase-variable experimentally (Jordan *et al.*, 2005). Transcriptional and translational phase variation have been extensively studied in the species of the genus *Neisseria*, however an additional class of simple sequence repeat-mediated phase variation has been described in *Helicobacter canadensis *following whole-genome analysis (Snyder *et al.*, 2010). Simple sequence repeat-mediated changes in the presence or absence of C-terminal cell wall attachment motifs has also been described in *Streptococcus agalactiae *(Janulczyk *et al.*, 2010). In *N. meningitidis*, a gene fusion between *pglB*2 and the downstream phosphoglycosyltransferase gene appears to be mediated by a poly-A repeat tract (Viburiene *et al.*, 2013).

With the availability of additional gonococcal genome sequences, the gonococcal phase-variable repertoire has here been re-assessed. As a result, phase variation in which repeats at the 3′ ends of genes mediate changes in the C-terminal sequence of the proteins is described as part of a refined phase-variable gene repertoire.

## Methods

### Identification of phase-variable genes.

Using the previous phase-variable gene repertoires reported for *N. gonorrhoeae* and *N. meningitidis *(Snyder *et al.*, 2001; Martin *et al.*, 2003; Jordan *et al.*, 2005), the homologues in *N. gonorrhoeae* strain NCCP11945 were sought (CP00150.1; Chung *et al.*, 2008). In addition, pattern search in *x*BASE (Chaudhuri *et al.*, 2008) was used to identify other repeats, based on previous evidence of phase variation in the species of the genus *Neisseria*: ≥ (G)8; ≥ (C)8; ≥ (CAAACAC)3; ≥ (CAAATAC)3; ≥ (CCCAA)3; ≥ (GCCA)3; ≥ (A)9; ≥ (T)9; ≥ (AAGC)3; ≥ (TTCC)3; and ≥ (CTTCT)3. No other repeats have been demonstrated to cause phase variation in this species. Genome sequences for *N. gonorrhoeae *strains NCCP11945 (NC_011035.1), FA1090 (NC_002946.2), FA19 (NZ_CP012026.1), FA6140 (NZ_CP012027.1), 35/02 (NZ_CP012028.1), and MS11 (NC_022240.1) were downloaded on 15th^th^April 2016 and compared using progressive Mauve v2.3.1 (Darling *et al.*, 2004) to identify orthologues (Table S1, available in the online Supplementary Material).

### Identification of repeat variation within *N. gonorrhoeae* strain NCCP11945.

*N. gonorrhoeae *strain NCCP11945 was grown on GC agar (Oxoid) with Kellogg’s (Kellogg *et al.*, 1963) and 5 % Fe(NO_3_)_3 _supplements at 37 °C in a candle tin for a period of 8 weeks with passages to fresh agar plates every 2 days or at 37 °C 5 % CO_2_ for a period of 20 weeks with passages to fresh agar plates every 2–3 days. At each passage, cells were scraped from the plate and resuspended in 1 ml of GC broth to a turbidity equivalent to a 0.5 McFarland standard before inoculation onto fresh plates using a sterile cotton swab. DNA was extracted from such resuspensions using the Puregene Yeast/Bacterial kit (Qiagen). A sample (1 µg or 100 ng) of the DNA was genome sequenced using the Ion Personal Genome Machine, Ion Express Fragment Library kit, Ion Express Template kit, and Ion Sequencing kit (Life Technologies) or using the Illumina-based methods of the MicrobesNG service (microbesng.uk). Sequence read data was interpreted using Galaxy on usegalaxy.org (Afgan *et al.*, 2016). Briefly, the reference sequence (NC_011035.1), Ion Torrent data for eight-week passages (KU1-4, KU1-45), Ion Torrent data for 20-week passages (KU1-95, KU1-96), and Illumina data for 20-week passages (2928-NS1_1 & 2929-NS1_2 and 2929-NS2_1 & 2929-NS2_2) were uploaded to Galaxy. The Ion Torrent bam format files were converted to fastq format using BAMTools Convert (Barnett *et al.*, 2011). FASTQ Groomer was used on all NGS data (Blankenberg *et al.*, 2010). Bowtie2 was used to map the reads against the reference (Langmead *et al.*, 2009; Langmead *et al.*, 2012) before visualisation using the Integrated Genomics Viewer (Robinson *et al.*, 2011; Thorvaldsdóttir *et al.*, 2013).

## Results and Discussion

### Phase variable genes

The phase-variable gene repertoire of *N. gonorrhoeae* strain NCCP11945 was investigated and compared against gonococcal strains FA1090, FA19, FA6140, 35/02, and MS11 to assess the presence of similar repeat tracts across the species and variations in repeat tract lengths between strains.

**Fig. 1.**
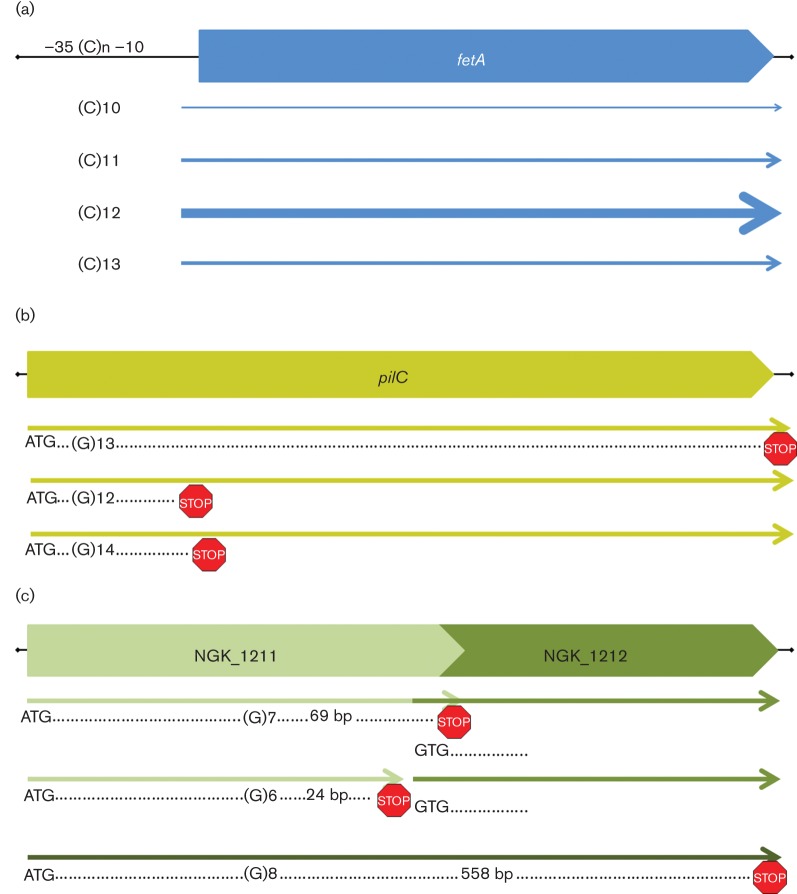
Illustrations of the types of phase variation in *N. gonorrhoeae*. (a): Transcriptional phase variation, in which changes in a repeat tract alter the facing and spacing of the −10 and −35 promoter elements and the level of transcription of the gene. Phase variation of *fetA* is used as an example, where it has been shown that differences in spacing of the −10 and −35 elements due to changes in the poly-C repeat tract alter expression levels, represented by the widths of the arrows (Carson *et al.*, 2000). (b): Translational phase variation, in which changes in a repeat tract towards the 5′ end of the coding sequence alter the reading frame of a coding region and switch expression on and off due to frame-shift. Phase variation of *pilC* is used as an example, where it has been shown that changes in the poly-G repeat tract generate frame-shifts which switch protein expression on and off (Jonsson *et al.*, 1991). (c): C-terminal phase variation, in which changes in a repeat tract towards the 3′ end of the coding sequence alter the reading frame of a coding region and switch the encoded C-terminal amino acids between the three reading frames. In the example NGK_1211, two of the reading frames result in different C-terminal ends to the protein, while the third generates a fusion with the downstream coding sequence, NGK_1212. Only some examples of C-terminal phase variation result in this type of fusion (Table 3).

Transcriptional phase variation is mediated by repeats within or associated with the promoter region (Fig. 1a). Changes in the repeat alters the level of transcription of the gene, as in *fetA (frpB*; NGK_2557) where differences in the length of the poly-C homopolymeric tract between the −10 and −35 promoter regions alters expression (Carson *et al.*, 2000). There are three transcriptional phase-variable genes in *N. gonorrhoeae *strain NCCP11945 (Table 1), *fetA *(NGK_2557), a lipoprotein (NGK_2186), and *porA *(NGK_0906/NGK_0907), yet in gonococci *porA* does not have an intact coding region. Variation in the repeats between gonococcal strains is found for all three transcriptional phase-variable genes (Table 1).

**Table 1.** Transcriptional phase variable genes in *N. gonorrhoeae*

Gene	FA1090 locus*	Repeat in FA1090*	NCCP11945 locus†	Repeat in NCCP11945†	Repeat in FA19‡	Repeat in FA6140§	Repeat in 35/02||	Repeat in MS11¶	*N. gonorrhoeae* candidacy#	Reference
*porA*	NGO_04715	(T)11C(G)6T	NGK_0906 & NGK_0907**	(T)9C(G)6T	(T)8C(G)8T	(T)9C(G)6T	(T)9C(G)6T	(T)9C(G)6TT	Known	van der Ende *et al.* (1995)
Lipoprotein	NGO2047	(A)9	NGK_2186††	(A)8	(A)8	(A)9	(A)9	(A)8	Yes	
*fetA / frpB*	NGO2093	(C)13	NGK_2557	(C)14	(C)10	(C)11	(C)11	(C)11	Known	Carson *et al.* (2000)

*From the *N. gonorrhoeae* strain FA1090 genome sequence (NC_002946.2).

†From the *N. gonorrhoeae* strain NCCP11945 genome sequence (CP00150.1).

‡From the *N. gonorrhoeae* strain FA19 genome sequence (NZ_CP012026.1).

§From the *N. gonorrhoeae* strain FA6140 genome sequence (NZ_CP012027.1).

||From the *N. gonorrhoeae* strain 35/02 genome sequence (NZ_CP012028.1).

¶From the *N. gonorrhoeae* strain MS11 genome sequence (NC_022240.1).

#Gene phase variation candidacy in *N. gonorrhoeae*. Known, phase variation has been reported in the literature. Yes, there is evidence of repeat tract variation between strains supporting phase variation.

**This coding sequenc appears to be frame-shifted and annotated as two coding sequences.

††NGK_2186 and NGO2047 annotations are on opposite strands.

Most common in the species of the genus *Neisseria* is translational phase-variation where, as in *pilC*, the repeat is within the 5′ portion of the coding region of the gene (Fig. 1b). Changes in the repeat tract generate frame-shift mutations in two of the three open reading frames, with the gene only being translated into protein when the repeat tract length puts the gene in-frame. Whilst many phase-variable genes in the species of the genus *Neisseria* contain homopolymeric tracts, some experience copy number changes in repetitive sequences, such as the CTTCT repeat in *opa *(Muralidharan *et al.*, 1987; Bhat *et al.*, 1991) or the AAGC repeat in *autA* (Peak *et al.*, 1999; Arenas *et al.*, 2015). In the *N. gonorrhoeae *strains examined here, the AAGC repeat in *virG* (NGK_0804) is only present in two or three copies (Table 2), rather than several copies as in NGK_0831a and *autA* (NGK_2082). Although *virG *has low copy number for the repeat, variations between strains are observed and strains with many copies may yet be identified [there are currently none >(AAGC)3 in the NCBI nr/nt or wgs databases], therefore it is placed amongst the phase-variable genes even though this may be at low frequency or be a strain-specific effect. There are 36 translational phase-variable genes in *N. gonorrhoeae *based on the species examined (Table 2).

**Table 2.** Translational phase-variable genes in *N. gonorrhoeae*

Gene	FA1090 locus*	Repeat in FA1090*	NCCP11945 locus†	Repeat in NCCP11945†	Repeat in FA19‡	Repeat in FA6140§	Repeat in 35/02||	Repeat in MS11¶	*N. gonorrhoeae* candidacy#	Reference
*pilC2*	NGO0055	(G)13	NGK_0074	(G)9	(G)10	(G)13	(G)9CAGG	(G)12	Known	Jonsson *et al.*, 1991
*opa*	NGO0066a	(CTTCT)13CTTCG	NGK_0096	(CTTCT)8CTTCC	(CTTCT)4CTTCC	CTT(CTTCT)10CTTCC	(CTTCT)6(CT TCC)2	(CTTCT)8CTT CC	Known	Stern & Meyer, 1987
*opa*	NGO0070	(CTTCT)9CTTCG	NGK_0102	(CTTCT)7CTTCC	(CTTCT)8CTTCC	(CTTCT)17CTTCC	(CTTCT)7CTT CC	(CTTCT)7CTT CC	Known	Stern & Meyer, 1987
*pglH*	NGO0086	(C)10	np	np	no repeat	no repeat	no repeat	np	Known	Power *et al.*, 2003
*pglG*	NGO0087	A(C)7	np	np	A(C)9	A(C)6	A(C)6	np	Known	Power *et al.*, 2003
*pglE*	NGO0207	(CAAACAC)4	NGK_0339	(CAAACAC)8 (CAAATAC)3	(CAAACAC)6CAAATACCAAACACCAAATAC	(CAAACAC)10CAAATACCAAACACCAAATACCAAACAC(CAAATAC)2	(CAAACAC)24 CAAATAC	(CAAACAC)15(CAAATAC)3	Known	Power *et al.*, 2003
*hsdS*	NGO_02155	(G)7	NGK_0571	(G)7	(G)8	(G)9	(G)7	(G)7	Known	Adamczyk-Poplawska *et al.*, 2011
Hypothetical	NGO0527	(C)6A(C)9GC	NGK_1405	(C)6A(C)8GC	(C)7A(C)4T(C )3GC	(C)7A(C)4T(C)3GC	(C)11A(C)8GC GC	(C)6A(C)14GC C	Yes	
*modB*	NGO0545	(CCCAA)12	NGK_1384	(CCCAA)11	(CCCAA)12	(CCCAA)4	(CCCAA)11	(CCCAA)7	Known	Srikhanta *et al.*, 2009
Replication initiation factor	NGO_06135	(C)8TTATCTAACA(G)7	NGK_1957	(C)11TTATCTAACA(G)8	(C)7TTATCTAACA(G)7	(C)6TTATCT AACA(G)5	(C)11TTATCT AACA(G)8	(C)10TTATCT AACA(G)7	Yes	
*modA*	NGO0641	(GCCA)37	NGK_1272	(GCCA)18	(GCCA)24GTCA	(GCCA)24	(GCCA)19	(GCCA)24	Known	Srikhanta *et al.*, 2009
Replication initiation factor	NGO_06695	(C)8TTATCTAACA(G)7	NGK_1486	(C)9TTATCTAACA(G)7	(C)7TTATCTAACA(G)7	(C)9TTATCT AACA(G)6	(C)11TTATCT AACA(G)8	(C)9TTATCTA ACA(G)7	Yes	
*opa*	NGO0950a	(CTTCT)16CTTCC	NGK_0847	(CTTCT)19CTTCC	(CTTCT)13CTTCC	(CTTCT)16CTTCC	(CTTCT)8CTT CC	(CTTCT)4CTT CC	Known	Stern & Meyer, 1987
Hypothetical	NGO0964	(AAGC)4	NGK_0831a	(AAGC)8	(AAGC)15	(AAGC)7	(AAGC)9	(AAGC)7	Yes	
*virG*	NGO0985	(AAGC)3	NGK_0804	(AAGC)3	(AAGC)2	(AAGC)3	(AAGC)3	(AAGC)3	Yes	
*opa*	NGO1040a	(CTTCT)20CTTCC	NGK_0749	(CTTCT)20CTTCC	(CTTCT)10CTTCC	(CTTCT)7CTTCC	(CTTCT)12CT TCC	(CTTCT)13CT TCCCTTCT(C TTCC)2	Known	Stern & Meyer, 1987
*opa*	NGO1073a	(CTTCT)2CTTCC	NGK_0693	(CTTCT)10CTTCC	(CTTCT)11CTTCC	(CTTCT)12CTTCC	(CTTCT)18CT TCC	(CTTCT)7CTT CC	Known	Stern & Meyer, 1987
*opa*	NGO1277a	(CTTCT)11CTTCC	NGK_1495	(CTTCT)7CTTCC	CTT(CTTCT)11CTTCC	(CTTCT)11CTTCC	(CTTCT)7CTT CC	(CTTCT)8CTT CC	Known	Stern & Meyer, 1987
Adhesion	NGO1445	(CAAG)20CAAA	NGK_1705	(CAAG)12CAAA	(CAAG)12CAAA	(CAAG)6CAAA	(CAAG)9CAAA	(CAAG)6CAAA	Yes	
*opa*	NGO1463a	(CTTCT)10CTTCC	NGK_1729	(CTTCT)7CTTCC	(CTTCT)11CTTCC	(CTTCT)12CTTCC	(CTTCT)12CT TCC	(CTTCT)10CT TCC	Known	Stern & Meyer, 1987
*opa*	NGO1513	(CTTCT)12CTTCG	NGK_1799	(CTTCT)14CTTCC	CTT(CTTCT)10CTTCC	np	(CTTCT)6CTT CG	(CTTCT)7CTT CG	Known	Stern & Meyer, 1987
*opa*	NGO1553a	(CTTCT)4CTTCC	NGK_1847	(CTTCT)9CT TCC	(CTTCT)17CTTCC	(CTTCT)8CTTCC	(CTTCT)8CTT CC	(CTTCT)14CT TCC	Known	Stern & Meyer, 1987
*autA*	NGO1689	(AAGC)3	NGK_2082	(AAGC)3	(AAGC)14	(AAGC)3	(AAGC)3	(AAGC)3	Known	Peak *et al.*, 1999 ; Arenas *et al.*, 2015
*pgtA*	NGO1765	(G)11	NGK_2516	GGGAGCGGG	(G)19	(G)19	GGGAGCGGG	GGGAGCGGG	Known	Banerjee *et al.*, 2002
Repetitive large surface lipoprotein	NGO_09875 & NGO_09870**	(G)8	NGK_2422 & NGK_2423**	(G)7	(G)7	(G)7	(G)7	(G)7	Yes	
*opa*	NGO1861a	(CTTCT)13CTTCC	NGK_2410	(CTTCT)11CTTCC	(CTTCT)13CTTCC	(CTTCT)2CTTCC	(CTTCT)13CT TCC	(CTTCT)30CT TCC	Known	Stern & Meyer, 1987
*pilC1*	NGO1912	(G)11	NGK_2342	(G)13	(G)11	GGGC(G)11	(G)15	(G)11	Known	Jonsson *et al.*, 1991
Hypothetical	NGO1953	(C)8	NGK_2297	(C)8	(C)8	(C)8	(C)9	(C)8	Yes	
Pyrimidine 5′- nucleotidase	NGO2055 & NGO2054**	(C)6	NGK_2176	CAAACCCC	CAAACCCC	CAAACCCC	(C)9	(C)10	Yes	
*opa*	NGO2060a	(CTTCT)10CTTCG	np		np	np	(CTTCT)6CTT CC	(CTTCT)7CTT CC	Known	Stern & Meyer, 1987
*opa*	np		NGK_2170	(CTTCT)14CTTCC	np	np	np	np	Known	Stern & Meyer, 1987
*lgtG*	NGO2072	(C)11	NGK_2534 & NGK_2533**	(C)12	(C)10	(C)10	(C)10	(C)10	Known	Mackinnon *et al.*, 2002
*hpuA*	NGO2110	(G)9	NGK_2581	(G)10	(G)9	(G)9	(G)8	(G)8	Known	Chen *et al.*, 1998
*lgtA*	NGO11610	(G)11	NGK_2630	(G)11	(G)14A	(G)17A	(G)20A	(G)10A	Known	Erwin *et al.*, 1996
*lgtC*	NGO2156	(G)14	NGK_2632	(G)13	(G)13	(G)10	(G)16	(G)8	Known	Shafer *et al.*, 2002
*lgtD*	NGO2158	A(G)14	NGK_2634	A(G)16	(G)13	A(G)12	A(G)18	A(G)13	Known	Shafer *et al.*, 2002

*From the *N. gonorrhoeae* strain FA1090 genome sequence (NC_002946.2).

†From the *N. gonorrhoeae* strain NCCP11945 genome sequence (CP00150.1).

‡From the *N. gonorrhoeae* strain FA19 genome sequence (NZ_CP012026.1).

§From the *N. gonorrhoeae* strain FA6140 genome sequence (NZ_CP012027.1).

||From the *N. gonorrhoeae* strain 35/02 genome sequence (NZ_CP012028.1).

¶From the *N. gonorrhoeae* strain MS11 genome sequence (NC_022240.1).

#Gene phase variation candidacy in *N. gonorrhoeae*. Known , phase variation has been reported in the literature. Yes, there is evidence of repeat tract variation between strains supporting phase variation.

**This coding sequence appears to be frame-shifted and annotated as two coding sequences.

np, The coding sequence is not present in this strain.

In addition, a third class of repeat-mediated phase-variable gene was identified (Snyder *et al.*, 2010). In these C-terminal phase-variable genes, a repeat tract towards the 3′ of the coding region is able to alter the sequence at the C-terminus of the encoded protein (Fig. 1c). In *N. gonorrhoeae* strain NCCP11945, four of these C-terminal phase-variable genes were identified (Table 3). It is likely that in the case of the pilin sequence (NGK_2161), changes in the repeat are causing pilus protein changes, mediating antigenic variation through a phase-variable mechanism. Comparisons also show repeat tract variation in a membrane protein (NGK_1211) and *mafB *cassette (NGK_1624), supporting C-terminal phase variation in the species of the genus *Neisseria* Variations in the products of *mafB *cassettes are believed to contribute to competition between species within the niche (Jamet *et al.*, 2015). Although no variation was observed in these strains in *ispH *(NGK_0106), (G)8 repeats are known to vary in *lgtC *(NGK_1632), *hpuA *(NGK_2581), and *hsdS *(NGK_0571) (Table 2), therefore it is highly likely that the repeat in *ispH* also has the capacity to vary.

**Table 3.** C-terminal phase-variable genes in *N. gonorrhoeae*

Gene	FA1090 locus*	Repeat at in FA1090*	NCCP11945 locus†	Repeat in NCCP11945†	Amino acids after the repeat in each frame of NCCP11945‡‡‡			FA19§	FA6140||	35/02 ¶	MA11#	*N. gonorrhoeae* candidacy**
*ispH*	NGO0072	(G)8	NGK_0106	(G)8	26	50	61	(G)8	(G)8	(G)8	(G)8	(Yes)
Menbrane protein	NGO0691	(G)6	NGK_1211	(G)7	23	8	186^	(G)7	(G)6	(G)7	(G)7	Yes
*m afB *cassette	WX61_RS02820	(C)7	NGK_1624	(C)9	46	2	37	deletion††	(C)10	(C)8	(C)10	Yes
Pilin cassette	WX61_RS02820	CCGC	NGK_2161	(C)8	20	8	91¶	(C)5GCC	CCGCC	(C)5	CCT(C)5	Yes

*From the *N. gonorrhoeae* strain FA1090 genome sequence (NC_002946.2).

†From the *N. gonorrhoeae* strain NCCP11945 genome sequence (CP00150.1).

‡In each column are the number of amino acids encoded 3′ of the repeat before the closest termination codon in each of the three reading frames.

§From the *N. gonorrhoeae* strain FA19 genome sequence (NZ_CP012026.1).

||From the *N. gonorrhoeae* strain FA6140 genome sequence (NZ_CP012027.1).

¶From the *N. gonorrhoeae* strain 35/02 genome sequence (NZ_CP012028.1).

#From the *N. gonorrhoeae* strain MS11 genome sequence (NC_022240.1).

**Gene phase variation candidacy in *N. gonorrhoeae*. Yes, there is evidence of repeat tract variation between strains supporting phase variation. (Yes), although there is no variation between strains investigated here, tracts of this length vary in other genes (Chen *et al.*, 1998; Shafer *et al.*, 2002; Adamczyk-Poplawska *et al.*, 2011).

††There is a 400 bp deletion in this strain encompassing the region that would contain this repeat.

A number of previously reported candidates are not supported by evidence of phase variation, based on the absence of tract length changes between the strains (Table 4). For example, although tract variation was reported for *cvaA* (NGK_0168), *mafA*-3 (NGK_2270), and *dca* (NGK_1830) in *N. meningitidis* (Martin *et al.*, 2003), there are no changes observed in the short (C)4 and (G)5 tracts in these genes in *N. gonorrhoeae *(Table 4). They are therefore unlikely to be phase-variable in this species. Likewise, neither of the dinucleotide-repeat-containing genes (NGK_1607 and NGK_2274) show variations (Table 4); dinucleotides are not likely to be phase-variable in the species of the genus *Neisseria* (Martin *et al.*, 2003). All of these genes contain short repeats that do not vary or alternative nucleotide sequences in the strains investigated (Table 4).

**Table 4.** Genes for which there is no evidence of phase variation in *N. gonorrhoeae*

Gene	FA1090 locus*	Repeat in FA1090*	NCCP11945 locus†	Repeat in NCCP11945†	Repeat in FA19‡	Repeat in FA6140§	Repeat in 35/02||	Repeat in MS11¶	*N. gonorrhoeae* candidacy#
Prolyl endopeptidase	NGO0026	GGGGCGG	NGK_0034	GGGGCGG	GGGGCGG	GGGGCGG	GGGGCGG	GGGGCGG	No. Replacement tract
*pill/wbpC*	NGO0065	C(G)6	NGK_0089**	C(G)6	C(G)6	C(G)6	C(G)6	C(G)6	No. No variation.
Phosphoesterase	NGO0081	(C)7	NGK_0 n 9	(C)7	(C)7	(C)7	(C)7	(C)7	No. No variation.
Hypothetical	NGO0121	(A)6	NGK_0167	(A)6	(A)6	(A)6	(A)6	(A)6	No. No variation.
*cvaA*	NGO0123	(C)4	NGK_0168	(C)4	(C)4	(C)4	(C)4	(C)4	No. No variation.
*potD* -2	NGO0206	AA(C)5	NGK_0338	AA(C)5	AA(C)5	AA(C)5	AA(C)5	AA(C)5	No. No variation.
Hypothetical	NGO0532	AACCGGCAAACA	NGK_1400	AACCGGCAAACA	AACCGGCAAACA	AACCGGCAAACA	AACCGGCAAACA	AACCGGCAAACA	No. Replacement tract
*nifS*	NGO0636	CCACACCC	NGK_1278	CCACACCC	CCACACCC	CCACACCC	CCACACCC	CCACACCC	No. Replacement tract
*lldD*	NGO0639	(G)7	NGK_1275	(G)7	(G)7	(G)7	(G)7	(G)7	No. No variation.
Methylase NlalV	NGO0676	(A)9	NGK_1230	(A)9	(A)9	(A)9	(A)9	(A)9	No. No variation.
*dnaX*	NGO0743	(C)7	NGK_1135	(C)7	(C)7	(C)7	(C)7	(C)7	No. No variation.
*mobA*	NGO0754	GGAAGG	NGK_1123	GGAAGG	GGAAGG	GGAAGG	GGAAGG	GGAAGG	No. Replacement tract
*ppx*	NGO1041	(C)7	NGK_0745	(C)7	(C)7	(C)7	(C)7	**(0**7	No. No variation.
*fxP/ccoP*	NGO1371	(AT)5	NGK_1607	(AT)5	(AT)5	(AT)5	(AT)5	(AT)5	No. No variation.
Hypothetical	NGO1384	G(A)7	NGK_1622	(A)8	(A)8	(A)8	(A)8	G(A)7	No. No variation in length.
*pntA*	NGO1470	CCCTGCTGG	NGK_1735	CCCTGCTGG	CCCTGCTGG	CCCTGCTGG	CCCTGCTGG	CCCTGCTGG	No. Replacement tract
*amiC*	NGO1501	TTCGCCC	NGK_1783	TTCGCCC	TTCGCCC	TTCGCCC	TTCGCCC	TTCGCCC	No. Replacement tract
*dca*	NGO1540	TGTGGGGG	NGK_1830	TGTGGGGG	TGTGGGGG	TGTGGGGG	TGTGGGGG	TGTGGGGG	No. Replacement tract
*anmK*	NGO1583	(C)7	NGK_1884	(C)7	(C)7	(C)7	(C)7	(C)7	No. No variation.
*dinG*	NGO1708	(C)4T CC	NGK_2106	(C)4TCC	(C)4TCC	(C)4TCC	(C)4TCC	(C)4TCC	No. Replacement tract
*rplK*	NGO1855	(C)7	NGK_2416	(C)7	(C)7	(C)7	(C)7	(C)7	No. No variation.
Hypothetical	NGO1970	(TA)5	NGK_2274	(TA)5	(TA)5	(TA)5	(TA)5	(TA)5	No. No variation.
*mafA* -3	NGO1972	(G)5	NGK_2270	(G)5	(G)5	(G)5	(G)5	(G)5	No. No variation.
*map*	NGO1983	(C)6	NGK_2258	(C)6	(C)6	(C)6	(C)6	(C)6	No. No variation.
*plsX*	NGO2171	(TTCC)3	NGK_2652	(TTCC)3	(TTCC)3	(TTCC)3	(TTCC)3	(TTCC)3	No. No variation.
*lbpA*	NGO0260a	nr	NGK_0401	GGGGGCGG	GGGGGCGG	nr	nr	TGAAACGG	No. Replacement tract

*From the *N. gonorrhoeae* strain FA1090 genome sequence (NC_002946.2).

†From the *N. gonorrhoeae* strain NCCP11945 genome sequence (CP00150.1).

‡From the *N. gonorrhoeae* strain FA19 genome sequence (NZ_CP012026.1).

§From the *N. gonorrhoeae* strain FA6140 genome sequence (NZ_CP012027.1).

||From the *N. gonorrhoeae* strain 35/02 genome sequence (NZ_CP012028.1).

¶From the *N. gonorrhoeae* strain MS11 genome sequence (NC_022240.1).

#Gene phase variation candidacy in *N. gonorrhoeae*. No. Replacement tract: due to the replacement of the repeat tract with other nucleotides, this is not phase-variable. No. No variation: due to no observed variation in the repeat tract, this is not phase-variable. No. No variation in length: due to the equal length tract in all strains, this is not phase-variable.

**This coding sequence contains a point mutation, which generates a premature termination codon.

nr, The region of the coding sequence containing the repeat tract does not have homology to the aligned region in these strains.

This analysis identified 29 genes that are known to be phase variable (Tables 1, 2), either in *N. gonorrhoeae *or *N. meningitidis* including 12 paralogues of *opa *(11 in each strain; Muralidharan *et al.*, 1987; Bhat *et al.*, 1991) and 17 other known phase-variable genes (Stern *et al.*, 1987; Jonsson *et al.*, 1991; Van der Ende *et al.*, 1995; Erwin *et al.*, 1996; Chen *et al.*, 1998; Peak *et al.*, 1999; Carson *et al.*, 2000; Banerjee *et al.*, 2002; Mackinnon *et al.*, 2002; Shafer *et al.*, 2002; Power *et al.*, 2003; Srikhanta *et al.*, 2009; Adamczyk-Poplawska *et al.*, 2011; Arenas *et al.*, 2015). Thirteen additional genes have variations in the repeat tracts when the six *N. gonorrhoeae *genome sequences are compared, one transcriptional, nine translational, and three C-terminal repeats. Based on homology and presence of conserved domains, these genes are believed to encode two replication initiation factors, an adhesion protein, a pyrimidine 5′-nucleotidase, two lipoproteins, two membrane proteins, two secreted proteins, and three hypothetical proteins (Tables 1–3).

Combined with the previous data on repeat variation within and between gonococcal strains and demonstration of phase variation (Sparling *et al.*, 1986; Yang *et al.*, 1996; Lewis *et al.*, 1999; Snyder *et al.*, 2001; Power *et al.*, 2003; Jordan *et al.*, 2005; Srikhanta *et al.*, 2009), a revised repertoire of 43 transcriptional (Table 1), translational (Table 2), and C-terminal (Table 3) phase-variable genes is proposed for *N. gonorrhoeae *as a species. This is fewer than previous predictions (76 in Jordan *et al.*, 2005) and thus far two-thirds (67 %, 29 out of 43) have been experimentally demonstrated to be phase-variable (Tables 1, 2). The additional 14 genes, 13 of which show strain-to-strain repeat variation, require additional investigation.

### Phase variable repeat copy number variation *in vitro*

Previously, for *H. canadensis*, 454 and Illumina genome sequence read data was used to support candidacy of phase-variable genes (Snyder *et al.*, 2010). In the present study, Ion Torrent and Illumina genome sequence read data from *N. gonorrhoeae *strain NCCP11945 that had been passaged in the laboratory for 8 weeks or for 20 weeks was analysed for changes to phase-variable repeats for the 14 genes for which there is no within-strain evidence of phase variation (Tables 1–3). Changes were observed in known phase-variable genes *pilC*1, *opa*, and *fetA*, suggesting that read data can support phase variability by demonstrating within-strain variation in tracts (Table 5). Of the 14 genes, only *virG *(NGK_0804) and the pyrimidine 5′-nucleotidase (NGK_2176) showed no changes in repeats (Table 5). Probably, the *virG* (AAGC)3 copy number is too low to vary, however there may be strains with greater copy number in which it would. Likewise, the poly-C repeat in NGK_2176 has been replaced with CAAACCCC in strain NCCP11945 and therefore would not be expected to phase vary in this strain, however phase variation is likely in strain MS11, for example.

**Table 5.** Genes for which there is sequencing-based evidence of phase variation in *N. gonorrhoeae *strain NCCP11945

Gene	FA1090 locus*	Repeat in FA1090*	NCCP11945 locus†	Repeat in NCCP11945†	Repeat in FA19‡	Repeat in FA6140§	Repeat in 35/02||	Repeat in MS11¶	* N. gonorrhoeae* candidacy#	Ion Torrent* *	Illumina††
*ispH*	NGO0072	(G)8	NGK_0106	(G)8	(G)8	(G)8	(G)8	(G)8	(yes)	Repeat varies	Repeat does not vary
*virG*	NGO0985	(AAGC)3	NGK_0804	(AAGC)3	(AAGC)2	(AAGC)3	(AAGC)3	(AAGC)3	Yes	Repeat does not vary	Repeat does not vary
Hypothetical	NGO0964	(AAGC)4	NGK_0831a	(AAGC)8	(AAGC)15	(AAGC)7	(AAGC)9	(AAGC)7	Yes	Repeat varies	Repeat varies
Membrane protein	NGO0691	(G)6	NGK_1211	(G)7	(G)7	(G)6	(G)7	(G)7	Yes	Repeat varies	Repeat does not vary
Hypothetical	NGO0527	(C)6A(C)9GC	NGK_1405	(C)6A(C)8GC	(C)7A(C)4T(C)3GC	(C)7A(C)4T(C)3GC	(C)11A(C)8GCGC	(C)6A(C)14GCC	Yes	Repeat varies	Repeat does not vary
Replication initiation factor	NGO_06695	(C)8TTATCTAACA(G)7	NGK1486	(C)9TTATCTAACA(G)7	(C)7TTATCTAACA(G)7	(C)9TTATCTAACA(G)6	(C)11TT ATCTAACA(G)8	(C)9T T AT CT AACA(G)7	Yes	Repeat varies	Repeat varies
*mafB * cassette	NGO1386	(C)7	NGK_1624	(C)9	{deletion}	(C)10	(C)8	(C)10	Yes	Repeat varies	Repeat varies
Adhesion	NGO1445	(CAAG)20CAAA	NGK_1705	(CAAG)12CAAA	(CAAG)12CAAA	(CAAG)6CAAA	(CAAG)9CAAA	(CAAG)6CAA A	Yes	Repeat varies	Repeat varies
Replication initiation factor	NGO_06135	(C)8TTATCTAACA(G)7	NGK_1957	(C)11TTATCTAACA(G)8	(C)7TTATCTAACA(G)7	(C)6TTATCTAACA(G)5	(C)11TTATCTAACA(G)8	(C)10T TAT C TAACA(G)7	Yes	Repeat varies	Repeat varies
Pilin cassette	NGO_11140	CCGC	NGK_2161	(C)8	(C)5GCC	CCGCC	(C)5	CCT (C)5	Yes	Repeat varies	Repeat varies
Pyrimidine 5′-nucleotidase	NGO2055 & NGO2054II	(C)6	NGK_2176	CAAACCCC	CAAACCCC	CAAACCCC	(C)9	(C)10	Yes	No repeat	No repeat
Lipoprotein	NGO2047	(A)9	NGK_2186Â§Â§	(A)8	(A)8	(A)9	(A)9	(A)8	Yes	Repeat varies	Repeat varies
Hypothetical	NGO1953	(C)8	NGK_2297	(C)8	(C)8	(C)8	(C)9	(C)8	Yes	Repeat varies	Repeat does not vary
Repetitive large surface lipoprotein	NGO_09875 & NGO_09870II	(G)8	NGK_2422 & NGK_2423||	(G)7	(G)7	(G)7	(G)7	(G)7	Yes	Repeat varies	Repeat does not vary
*fetA * / *frpB*	NGO2093	(C)13	NGK_2557	(C)14	(C)10	(C)11	(C)11	(C)11	Known	Repeat varies	Repeat varies
*pilC * 1	NGO1912	(G)11	NGK_2342	(G)13	(G)11	GGGC(G)11	(G)15	(G)11	Known	Repeat varies	Repeat varies
*opa*	NGO0950a	(CTTCT)16CTTCC	NGK_0847	(CTTCT)19CTTCC	(CTTCT)13CTTCC	(CTTCT)16CTTCC	(CTTCT)8CTTCC	(CT T CT )4CT TCC	Known	No reads through repeat	Repeat varies

*From the *N. gonorrhoeae* strain FA1090 genome sequence (NC_002946.2).

†From the *N. gonorrhoeae* strain NCCP11945 genome sequence (CP00150.1).

‡From the *N. gonorrhoeae* strain FA19 genome sequence (NZ_CP012026.1).

§From the *N. gonorrhoeae* strain FA6140 genome sequence (NZ_CP012027.1).

||From the *N. gonorrhoeae* strain 35/02 genome sequence (NZ_CP012028.1).

¶From the *N. gonorrhoeae* strain MS11 genome sequence (NC_022240.1).

#Gene phase variation candidacy in *N. gonorrhoeae*. Known, phase variation has been reported in the literature. Yes, there is evidence of repeat tract variation between strains supporting phase variation. (Yes), although there is no variation between strains investigated here, tracts of this length vary on other genes (Chen *et al.*, 1998; Shafer *et al.*, 2002; Adamczyk-Poplawska *et al.*, 2011).

**Based on Ion Torrent sequencing data from cultures grown with passage for 8 weeks and from cultures grown with passage for 20 weeks (accession numbers SRR3547950, SRR3547951, SRR3547952, SRR3547953).

††Based on Illumina sequencing data from cultures grown with passage for 20 weeks (accession numbers SRR3547954, SRR3547955, SRR3547956, SRR3547957).

‡‡There is a 400 bp deletion in this strain encompassing the region that would contain this repeat.

§§NGK_2186 and NGO2047 annotations are on opposite strands.

||||This coding sequence appears to be frame-shifted and annotated as two coding sequences.

The Ion Torrent sequencing technology has been criticised for generating homopolymer-associated indels (Loman *et al.*, 2012) and that the tracts can be incorrect at more than eight bases (Quail *et al.*, 2012), the optimal length for phase variation. Homopolymeric tracts in Illumina data are believed to be less error prone (Schirmer *et al.*, 2015). However, repeat sequence data from Illumina often agreed with Ion Torrent on the presence of variation (9 of 14 genes with variation in Ion Torrent, Table 5). When the Illumina data did not show repeat variation, this often corresponded to relatively low read coverage of the region compared to the Ion Torrent data (Table S2).

It is currently impossible to differentiate genuine biologically induced indels from sequencing errors (Narzisi & Schatz, 2015). We may find that what we ascribe to errors can also be subtle changes that are constantly being generated within the bacterial population. From this data, the expected biological variation supporting phase variation appears to be present in *N. gonorrhoeae* strain NCCP11945 for 12 as yet unexplored genes.

## Conclusion

In conclusion, *N. gonorrhoeae *possesses three different mechanisms for phase variation: transcriptional; translational; and C-terminal. Stochastic systems obviously play important roles in the biology of the organism given the variety and number of genes involved. The functions of previously unexplored phase-variable genes, including one transcriptional phase-variable gene, nine translational phase-variable genes, and four C-terminal phase-variable genes require further investigation.

## Supplementary Data

26 Supplementary File 1

## References

[R1] Adamczyk-Poplawska M. Lower M. Piekarowicz A.(2011). Deletion of one nucleotide within the homonucleotide tract present in the *hsdS* gene alters the DNA sequence specificity of type I restriction-modification system NgoAV. J Bacteriol 193 6750–6759. 10.1128/JB.05672-11 21984785 PMC3232900

[R2] Afgan E. Baker D. Van den Beek M. Blankenberg D. Bouvier D. Čech M. Chilton J. Clements D. Coraor N.(2016). The Galaxy platform for accessible, reproducible and collaborative biomedical analyses: 2016 update. Nucleic Acids Res 44 W3–W10. 10.1093/nar/gkw343 27137889 PMC4987906

[R3] Arenas J. Cano S. Nijland R. Van Dongen V. Rutten L. Van der Ende A. Tommassen J.(2015). The meningococcal autotransporter AutA is implicated in autoaggregation and biofilm formation. Environ Microbiol 17 1321–1337. 10.1111/1462-2920.12581 25059714

[R4] Banerjee A. Wang R. Supernavage S. L. Ghosh S. K. Parker J. Ganesh N. F. Wang P. G. Gulati S. Rice P. A.(2002). Implications of phase variation of a gene (*pgtA*) encoding a pilin galactosyl transferase in gonococcal pathogenesis. J Exp Med 196 147–162. 10.1084/jem.20012022 12119340 PMC2193922

[R5] Barnett D. W. Garrison E. K. Quinlan A. R. Strömberg M. P. Marth G. T.(2011). BamTools: a C++ API and toolkit for analyzing and managing BAM files. Bioinformatics 27 1691–1692. 10.1093/bioinformatics/btr174 21493652 PMC3106182

[R6] Bhat K. S. Gibbs C. P. Barrera O. Morrison S. G. Jähnig F. Stern A. Kupsch E. M. Meyer T. F. Swanson J.(1991). The opacity proteins of *Neisseria gonorrhoeae* strain MS11 are encoded by a family of 11 complete genes. Mol Microbiol 5 1889–1901. 10.1111/j.1365-2958.1991.tb00813.x 1815562

[R7] Blankenberg D. Gordon A. Von Kuster G. Coraor N. Taylor J. Nekrutenko A. Galaxy Team(2010). Manipulation of FASTQ data with Galaxy. Bioinformatics 26 1783–1785. 10.1093/bioinformatics/btq281 20562416 PMC2894519

[R8] Bolotin D. A. Mamedov I. Z. Britanova O. V. Zvyagin I. V. Shagin D. Ustyugova S. V. Turchaninova M. A. Lukyanov S. Lebedev Y. B. Chudakov D. M.(2012). Next generation sequencing for TCR repertoire profiling: platform-specific features and correction algorithms. Eur J Immunol 42 3073–3083. 10.1002/eji.201242517 22806588

[R9] Bragg L. M. Stone G. Butler M. K. Hugenholtz P. Tyson G. W.(2013). Shining a light on dark sequencing: characterising errors in Ion torrent PGM data. PLoS Comput Biol 9 e1003031. 10.1371/journal.pcbi.1003031 23592973 PMC3623719

[R10] Carbonnelle E. Hill D. J. Morand P. Griffiths N. J. Bourdoulous S. Murillo I. Nassif X. Virji M.(2009). Meningococcal interactions with the host. Vaccine 27 B78–89. 10.1016/j.vaccine.2009.04.069 19481311

[R11] Carson S. D. Stone B. Beucher M. Fu J. Sparling P. F.(2000). Phase variation of the gonococcal siderophore receptor FetA. Mol Microbiol 36 585–593. 10.1046/j.1365-2958.2000.01873.x 10844648

[R12] Chaudhuri R. R. Loman N. J. Snyder L. A. Bailey C. M. Stekel D. J. Pallen M. J.(2008). xBASE2: a comprehensive resource for comparative bacterial genomics. Nucleic Acids Res 36 D543–546. 10.1093/nar/gkm928 17984072 PMC2238843

[R13] Chen C. J. Elkins C. Sparling P. F.(1998). Phase variation of hemoglobin utilization in *Neisseria gonorrhoeae*. Infect Immun 66 987–993. 9488386 10.1128/iai.66.3.987-993.1998 PMC108006

[R14] Chung G. T. Yoo J. S. Oh H. B. Lee Y. S. Cha S. H. Kim S. J. Yoo C. K.(2008). Complete genome sequence of *Neisseria gonorrhoeae* NCCP11945. J Bacteriol 190 6035–6036. 10.1128/JB.00566-08 18586945 PMC2519540

[R15] Darling A. C. Mau B. Blattner F. R. Perna N. T.(2004). Mauve: multiple alignment of conserved genomic sequence with rearrangements. Genome Res 14 1394–1403. 10.1101/gr.2289704 15231754 PMC442156

[R17] Erwin A. L. Haynes P. A. Rice P. A. Gotschlich E. C.(1996). Conservation of the lipooligosaccharide synthesis locus *lgt *among strains of *Neisseria gonorrhoeae*: requirement for *lgtE* in synthesis of the 2C7 epitope and of the β chain of strain 15253. J Exp Med 184 1233–1241. 10.1084/jem.184.4.1233 8879194 PMC2192810

[R18] Jamet A. Jousset A. B. Euphrasie D. Mukorako P. Boucharlat A. Ducousso A. Charbit A. Nassif X.(2015). A new family of secreted toxins in pathogenic *Neisseria* species. PLoS Pathog 11 e1004592. 10.1371/journal.ppat.1004592 25569427 PMC4287609

[R19] Janulczyk R. Masignani V. Maione D. Tettelin H. Grandi G. Telford J. L.(2010). Simple sequence repeats and genome plasticity in *Streptococcus agalactiae*. J Bacteriol 192 3990–4000. 10.1128/JB.01465-09 20494995 PMC2916363

[R20] Jonsson A. B. Nyberg G. Normark S.(1991). Phase variation of gonococcal pili by frameshift mutation in *pilC*, a novel gene for pilus assembly. EMBO J 10 477–488. 1671354 10.1002/j.1460-2075.1991.tb07970.x PMC452669

[R21] Jordan P. W. Snyder L. A. Saunders N. J.(2005). Strain-specific differences in *Neisseria gonorrhoeae* associated with the phase variable gene repertoire. BMC Microbiol 5. 10.1186/1471-2180-5-21 15857514 PMC1097732

[R16] Kellogg D. S. Jr Peacock W. L. Jr Deacon W. E. Brown L. Pirkle D. I.(1963). *Neisseria gonorrhoeae* I. virulence genetically linked to clonal variation. J Bacteriol 85 1274–1279. 14047217 10.1128/jb.85.6.1274-1279.1963 PMC278328

[R22] Langmead B. Trapnell C. Pop M. Salzberg S. L.(2009). Ultrafast and memory-efficient alignment of short DNA sequences to the human genome. Genome Biol 10 R25. 10.1186/gb-2009-10-3-r25 19261174 PMC2690996

[R23] Langmead B. Salzberg S. L.(2012). Fast gapped-read alignment with Bowtie 2. Nat Methods 9 357–359. 10.1038/nmeth.1923 22388286 PMC3322381

[R24] Lewis L. A. Gipson M. Hartman K. Ownbey T. Vaughn J. Dyer D. W.(1999). Phase variation of HpuAB and HmbR, two distinct haemoglobin receptors of *Neisseria meningitidis* DNM2. Mol Microbiol 32 977–989. 10.1046/j.1365-2958.1999.01409.x 10361300

[R25] Loman N. J. Misra R. V. Dallman T. J. Constantinidou C. Gharbia S. E. Wain J. Pallen M. J.(2012). Performance comparison of benchtop high-throughput sequencing platforms. Nat Biotechnol 30 434–439. 10.1038/nbt.2198 22522955

[R26] Mackinnon F. G. Cox A. D. Plested J. S. Tang C. M. Makepeace K. Coull P. A. Wright J. C. Chalmers R. Hood D. W.(2002). Identification of a gene (*lpt*-3) required for the addition of phosphoethanolamine to the lipopolysaccharide inner core of *Neisseria meningitidis* and its role in mediating susceptibility to bactericidal killing and opsonophagocytosis. Mol Microbiol 43 931–943. 10.1046/j.1365-2958.2002.02754.x 11929543

[R27] Martin P. Van de Ven T. Mouchel N. Jeffries A. C. Hood D. W. Moxon E. R.(2003). Experimentally revised repertoire of putative contingency loci in *Neisseria meningitidis* strain MC58: evidence for a novel mechanism of phase variation. Mol Microbiol 50 245–257. 10.1046/j.1365-2958.2003.03678.x 14507378

[R28] Moxon R. Bayliss C. Hood D.(2006). Bacterial contingency loci: the role of simple sequence DNA repeats in bacterial adaptation. Annu Rev Genet 40 307–333. 10.1146/annurev.genet.40.110405.090442 17094739

[R29] Muralidharan K. Stern A. Meyer T. F.(1987). The control mechanism of opacity protein expression in the pathogenic *Neisseriae*. Antonie Van Leeuwenhoek 53 435–440. 10.1007/BF00415499 3130782

[R30] Narzisi G. Schatz M. C.(2015). The challenge of small-scale repeats for indel discovery. Front Bioeng Biotechnol 3 8. 10.3389/fbioe.2015.00008 25674564 PMC4306302

[R31] Omer H. Rose G. Jolley K. A. Frapy E. Zahar J. R. Maiden M. C. Bentley S. D. Tinsley C. R. Nassif X. Bille E.(2011). Genotypic and phenotypic modifications of *Neisseria meningitidis* after an accidental human passage. PLoS One 6 e17145. 10.1371/journal.pone.0017145 21386889 PMC3046118

[R32] Peak I. R. Jennings M. P. Hood D. W. Moxon E. R.(1999). Tetranucleotide repeats identify novel virulence determinant homologues in *Neisseria meningitidis*. Microb Pathog 26 13–23. 10.1006/mpat.1998.0243 9973577

[R33] Power P. M. Roddam L. F. Rutter K. Fitzpatrick S. Z. Srikhanta Y. N. Jennings M. P.(2003). Genetic characterization of pilin glycosylation and phase variation in *Neisseria meningitidis*. Mol Microbiol 49 833–847. 10.1046/j.1365-2958.2003.03602.x 12864863

[R34] Quail M. A. Smith M. Coupland P. Otto T. D. Harris S. R. Connor T. R. Bertoni A. Swerdlow H. P. Gu Y.(2012). A tale of three next generation sequencing platforms: comparison of Ion Torrent, Pacific Biosciences and Illumina MiSeq sequencers. BMC Genomics 13 341. 10.1186/1471-2164-13-341 22827831 PMC3431227

[R35] Robinson J. T. Thorvaldsdóttir H. Winckler W. Guttman M. Lander E. S. Getz G. Mesirov J. P.(2011). Integrative genomics viewer. Nat Biotechnol 29 24–26. 10.1038/nbt.1754 21221095 PMC3346182

[R36] Schirmer M. Ijaz U. Z. D'Amore R. Hall N. Sloan W. T. Quince C.(2015). Insight into biases and sequencing errors for amplicon sequencing with the Illumina MiSeq platform. Nucleic Acids Res 43 e37. 10.1093/nar/gku1341 25586220 PMC4381044

[R37] Shafer W. M. Datta A. Kolli V. S. Rahman M. M. Balthazar J. T. Martin L. E. Veal W. L. Stephens D. S. Carlson R.(2002). Phase variable changes in genes *lgtA* and *lgtC* within the *lgtABCDE* operon of *Neisseria gonorrhoeae* can modulate gonococcal susceptibility to normal human serum. J Endotoxin Res 8 47–58. 10.1177/09680519020080010501 11981445

[R38] Snyder L. A. Butcher S. A. Saunders N. J.(2001). Comparative whole-genome analyses reveal over 100 putative phase-variable genes in the pathogenic *Neisseria* spp. Microbiology 147 2321–2332. 10.1099/00221287-147-8-2321 11496009

[R39] Snyder L. A. Loman N. J. Linton J. D. Langdon R. R. Weinstock G. M. Wren B. W. Pallen M. J.(2010). Simple sequence repeats in *Helicobacter canadensis* and their role in phase variable expression and *C*-terminal sequence switching. BMC Genomics 11. 10.1186/1471-2164-11-67 20105305 PMC2823697

[R40] Sparling P. F. Cannon J. G. So M.(1986). Phase and antigenic variation of pili and outer membrane protein II of *Neisseria gonorrhoeae*. J Infect Dis 153 196–201. 10.1093/infdis/153.2.196 2418125

[R41] Srikhanta Y. N. Dowideit S. J. Edwards J. L. Falsetta M. L. Wu H. J. Harrison O. B. Fox K. L. Seib K. L. Maguire T. L.(2009). Phasevarions mediate random switching of gene expression in pathogenic *Neisseria*. PLoS Pathog 5 e1000400. 10.1371/journal.ppat.1000400 19390608 PMC2667262

[R42] Stern A. Meyer T. F.(1987). Common mechanism controlling phase and antigenic variation in pathogenic neisseriae. Mol Microbiol 1 5–12. 10.1111/j.1365-2958.1987.tb00520.x 2455211

[R43] Thorvaldsdóttir H. Robinson J. T. Mesirov J. P.(2013). Integrative genomics viewer (IGV): high-performance genomics data visualization and exploration. Brief Bioinform 14 178–192. 10.1093/bib/bbs017 22517427 PMC3603213

[R46] Van der Ende A. Hopman C. T. Zaat S. Essink B. B. Berkhout B. Dankert J.(1995). Variable expression of class 1 outer membrane protein in *Neisseria meningitidis* is caused by variation in the spacing between the −10 and −35 regions of the promoter. J Bacteriol 177 2475–2480. 7730280 10.1128/jb.177.9.2475-2480.1995 PMC176907

[R44] Viburiene R. Vik Å. Koomey M. Børud B.(2013). Allelic variation in a simple sequence repeat element of neisserial *pglB2* and its consequences for protein expression and protein glycosylation. J Bacteriol 195 3476–3485. 10.1128/JB.00276-13 23729645 PMC3719539

[R45] Yang Q. L. Gotschlich E. C.(1996). Variation of gonococcal lipooligosaccharide structure is due to alterations in poly-G tracts in *lgt* genes encoding glycosyl transferases. J Exp Med 183 323–327. 10.1084/jem.183.1.323 8551240 PMC2192423

